# Development of a New Electronic Death Certificate and Death Management Module Integrated Into the Health Information System of a Tertiary Hospital in Mali: Implementation Report

**DOI:** 10.2196/62949

**Published:** 2025-09-18

**Authors:** Mahmoud Cissé, Mahamoudane Niang, Abdrahamane Anne, Mariam Sidibé, Dramane Traoré, Abdoulaye Traoré, Idrissa Traoré, Cheick Oumar Bagayoko

**Affiliations:** 1 Center for Expertise and Research in Telemedicine and E-Health - CERTES, Bamako, Mali; 2 Department of Study and Research in Public Health (DERSP), Faculty of Medicine and Odontostomatology (FMOS), University of Sciences, Techniques and Technologies of Bamako, Bamako, BP 1805, Mali, +223 74014435; 3 Mother and Child Hospital, Bamako, Mali

**Keywords:** death certificate, hospital Information Systems, international classification of diseases, vital statistics, digital health

## Abstract

**Background:**

Death certification provides reliable epidemiological data that are essential for public health decision-making. Implementing an electronic death certificate improves data quality, accuracy, and timeliness. Recognizing its importance, we developed and integrated a mortality management module into the hospital “Le Luxembourg,” enhancing mortality data collection and utilization.

**Objective:**

This implementation aimed to improve the completeness and accuracy of mortality data, accelerate the timeliness of death reporting, and facilitate the downstream use of data to inform public health planning and research.

**Methods:**

We began by analyzing the existing infrastructure and organizational setup within the hospital. Through a series of interviews, we identified the needs of all users, enabling the design of a module suited to all levels of operation. We implemented a module comprising multiple functionalities, including certificate editing, validation, storage, mortality statistics generation. It also integrates *ICD-10* coding and follows the World Health Organization (WHO) model, while remaining adapted to the hospital’s specific context. To ensure optimal usability, we assembled a project team that included a mortality audit committee. After implementation, all users received training and continuous direct technical support was provided.

**Results:**

We developed a new death certificate model in line with WHO recommendations. Access to the certificate is secured by a unique username and password. To improve data quality, the certification process involves several validation steps: initial recording, which can be modified when the medical section is not completed by a senior physician; pre-validation by the senior physician and final validation by the mortality audit committee. The chain of morbid events is documented using *ICD-10* diagnoses. Beyond the certificate itself, the system also allows for civil registration of the death. Moreover, the module can generate statistics based on multiple criteria. This process takes place with the involvement and active engagement of all stakeholders.

**Conclusions:**

We established a unique, secure, WHO-compliant death certificate model that ensures high-quality, easily exploitable, and well-archived data. The experience of this hospital may serve as a foundation for scaling up this model to other healthcare facilities within the country.

## Introduction

### Background

Knowledge of the causes of mortality is an integral part of the foundation of public health policies, strategies and programs [1]. Death certification plays a central role in the collection of demographic and epidemiological data worldwide. Not only does it identify the causes of mortality, it is also a fundamental tool for guiding public health policies.

For several years now, high-income countries, notably in Europe and North America, have been deploying electronic death certification systems to improve the efficiency, accuracy and speed of the information collected [2,3]. For example, France introduced such a system in 2007 with the CertDc project to gradually replace traditional paper forms [3]. In Portugal, a similar system has been in place since 2013, having made a 100% successful transition from paper to electronic certification after 2 years.

The advantages of such a system are multiple. Firstly, digitizing the process eliminates manual transcription errors, but also speeds up data processing, as shown by a French study on the contribution of electronic certification [4].

In Africa, where the civil registry system is still largely dominated by traditional methods, the need for modernization is pressing. The United Nations Economic Commission for Africa (UNECA) report on death registration highlights that of the 46 World Health Organization (WHO) member states in the African region, only 1 can provide high-quality cause-of-death data (Mauritius), and 3 others can provide low- or medium-quality data (ie, South Africa, Seychelles and Zimbabwe) [5]. Nevertheless, we noted a few rare initiatives, such as Burkina Faso’s implementation of an electronic death certificate at Bobo University Hospital [6].

On the other hand, Mali like many other African countries, faces similar challenges, notably insufficient coverage of the civil registry system, delays in the transmission of paper death certificates and frequent errors in data collection.

These aspects reduce the quality of national and international mortality statistics and limits their value for health planning and health policy development [7]. Mali is no exception, despite its high maternal, infant, child, infant-child and neonatal mortality rates [8]

In Mali, death certificates vary across health facilities and even within the same hospital. Some services lack a standard form, relying on handwritten notes. This inconsistency limits mortality data availability and accuracy, with records often incomplete, containing only basic details like name, sex, date of death, and a vague diagnosis (eg, cardio-respiratory arrest). Archiving is neither systematic nor organized, and copies are not always retained. Deaths are often tracked by marking “Deceased” or “DCD” in patient registers.

To overcome these problems, we need adapted and harmonized methods and tools that comply with certain norms and standards, and the advantage of using digital tools in our context is no longer in doubt [8-10].

The hospital “Le Luxembourg” in Bamako initiated a system to digitize and integrate the death certificate into its Health Information System (HIS) to improve mortality data management. Despite its technical capacity and nationwide patient intake, it faced challenges similar to other health facilities in collecting and using mortality data. Recording 556 deaths annually, the hospital wants to develop an electronic death certificate for better archiving and data use, as detailed in this implementation report.

### Objectives

The implementation aimed to improve the completeness and accuracy of mortality data, accelerate the timeliness of death reporting, and facilitate the downstream use of data to inform public health planning and research.

## Methods

### Conception and Development

This project was part of a thesis internship, led by a student with support from the Center for Expertise and Research in Telemedicine and E-Health (CERTES), which maintains the HIS. Implemented at the hospital “Le Luxembourg,” it involved system analysis, requirement collection, and specifications drafting. Development took three months, carried out by two CERTES developers and the student.

For the successful implementation of the project, a team was established, composed of the head of the public health and medical informatics department of “Le Luxembourg” Hospital, the trainee, the developers of the Cinz@n (HIS), the users represented by doctors (ie, residents, general practitioners, and specialists), nurses from various departments, and the hospital registrar.

All users were involved from the design phase through interviews aimed at understanding their needs. Physicians participated in the validation of the medical component, while administrative staff contributed to the design of the printable certificate. Their involvement facilitated acceptance of the new process.

#### Analysis of the ongoing certification process and HIS

When a death occurs or is confirmed on arrival, a doctor or resident issues a death certificate in duplicate. One copy is provided to the family for administrative use, while the other serves as proof for body deposit and burial. The deceased’s name is recorded in consultation registers or a dedicated logbook, but no cause of death is documented.

At the end of each month, hospital staff manually count the number of deaths and submit a monthly activity report to the HIS officer, who enters the data into DHIS2, a national HIS. However, this report lacks patient demographics and cause-of-death details.

The hospital operates Cinz@n, a PHP/MySQL-based HIS that integrates patient records, consultations, hospitalization, pharmacy, and related data. However, it does not track death records.

#### New electronic certification process implemented

A new digital death certification system has been introduced to improve record accuracy and medical data storage. This system ensures that both administrative and medical information are electronically archived in Cinz@n for statistical purposes. The key actors in the certification process are: doctors and residents, nurses, civil registrar and death audit committee.

The module includes the following key functionalities:

- Generation of death certificates: This feature is composed of two main sections:

- An administrative section that records patient identity, hospital details, time of death, and burial information
- A medical section that digitally captures cause-of-death details, comorbidities, and relevant external factors

- Deceased patients list: a searchable registry of all recorded deaths within the facility
- Certificates pending preliminary validation: death certificates awaiting review and approval by a senior physician
- Certificates pending final validation: certificates that have not yet been approved by the audit committee
- Archived certificates: a secure and structured repository of validated death certificates
- Statistical dashboard: aggregated data on mortality trends by sex, age, hospital unit, causes of death, and more
- Configuration panel: allows customization of fields, labels, access rights, and other module parameters

**Figure 1.**
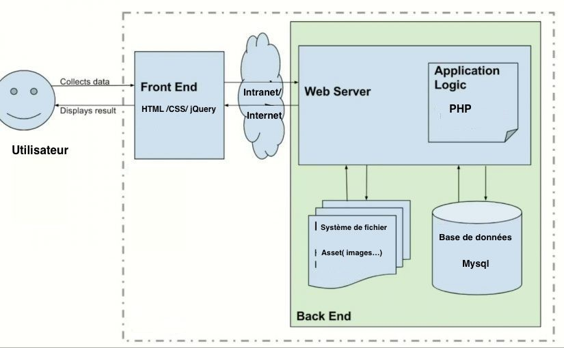
Architecture of the HIS “Cinz@n”. HIS: Health information system; PHP: Hypertext preprocessor.

The module architecture is based on that of the HIS “*Cinz@n* ” (Figure 1).

In terms of security, the server ensures security by recognizing devices using encrypted authentication, tracking access logs, and deactivating accounts after three failed attempts. A dedicated backup server ensures regular and secure data replication to prevent loss. User access is profile-based, ensuring that each stakeholder (ie, physicians, administrators, civil registry officers) can only access the information relevant to their responsibilities.

The module integrates International Classification of Diseases, Tenth Revision (*ICD-10)* for causes of death, consultations, and comorbidities, while Cinz@n uses the same patient ID and HL7 (Health Level Seven) standards, to facilitate the integration of this module into other systems.

A total of 66 users including 33 doctors, 26 resident doctors, 6 nurses, and 1 midwife, were trained to use the death module and code causes of death. The training consisted of lectures, practical tutorials, and WHO-provided exercises to enhance proficiency.

### Budget Report

The project required no additional budget as the hospital already had the necessary infrastructure. The module is maintained internally by the hospital’s medical informatics department, with updates managed by a trained technical focal point. With maintenance limited to software support, the solution is sustainable without external funding. Its design also allows for easy deployment in other hospitals using the same HIS.

### Ethical Considerations

This project, integrated into routine hospital activities, aimed only to digitalize the death certificate and enable the secure storage and structured use of mortality data. No sensitive or identifiable patient information was used, and no direct interaction with patients or their relatives occurred. In accordance with local guidelines, no ethical approval was required. Data confidentiality and security were strictly maintained.

This manuscript complies with the implementation reporting guidelines [11].

## Implementation (Results)

### Coverage and Stage

After the design and development phases, we implemented a new model of death certificate at the hospital *“*Le Luxembourg*,”* which included an administrative (Multimedia Appendix 1) and medical (Multimedia Appendix 2) component. This multifunctional death management module (Multimedia Appendix 3) is now integrated into the HIS. Before implementation, from a methodological standpoint, we adopted an iterative approach, starting with an initial prototype tested in one hospital department, which was then improved through several short cycles, incorporating user feedback at each stage.

User feedback at the end of each training session and two months postimplementation indicated a high level of satisfaction. This was especially attributed to the ease of access to patient information and the automatic generation of certificates.

**Figure 2.**
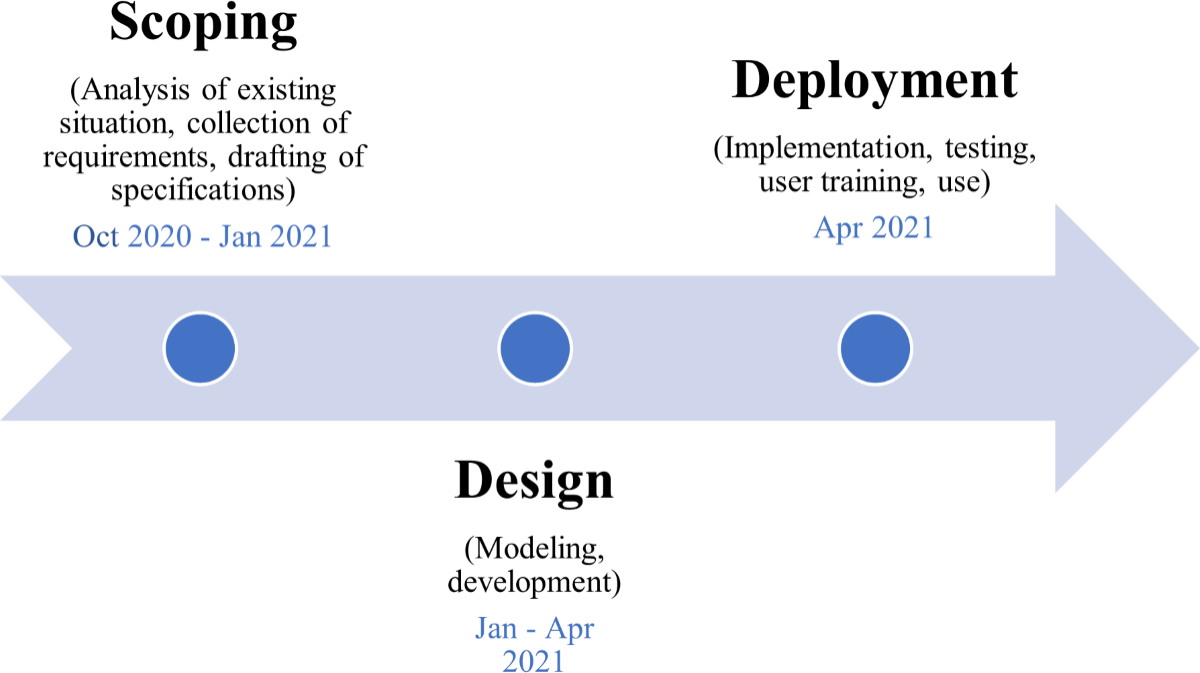
Project schedule.

The project timeline is presented in Figure 2.

To access the death management module, users require a username and password. Key features include creating death certificates, listing deceased patients, viewing certificates awaiting prevalidation and validation, accessing archived certificates, statistics, and settings.

Users can search for a patient using an ID number, name, or phone number before issuing a death certificate.

The “Establish a Death Certificate” menu integrates both administrative and medical components. Administrative data (Multimedia Appendix 4) are automatically retrieved from the patient’s record. The medical section (Multimedia Appendix 5) also pulls relevant information, including reason for admission and comorbidities, with *ICD-10* used for cause-of-death coding.

The validation process follows a structured approach. Automatic alerts remind doctors to correct pending certificates, and pop-ups confirm actions before validation. This validation process ensures data quality, with data being easily obtained and managed from the dedicated menu. Users can filter data and export reports in the Microsoft Excel format for further analysis.

A dedicated interface allows the civil registrar to access the administrative section of the certificate and process official death declarations. By searching the deceased’s ID number, the registrar records details of the declarant (ie, relative) before confirming the declaration. Once completed, the entry becomes inactive and validated.

Within three months of implementation, a total of 62 death certificates were recorded. Although the volume appears modest, the key value of the system lies in the structured storage and availability of comprehensive mortality data.

### Lessons Learned, Key Success Factors, and Failure

The success of the project was driven by strong leadership, particularly the involvement of key institutional figures such as the heads of the medical informatics department and the medical board. Integrating the module directly into the existing HIS, rather than creating a parallel system, greatly facilitated adoption. Initial resistance from users was overcome through training and support. The inclusion of *ICD-10* coding improved data quality and encouraged broader use of the system. Academic clinicians engaged early, motivated by access to structured data for research. The availability of IT infrastructure also played a critical role. However, limited staff numbers in departments hindered real-time data entry, underscoring the need to consider workforce constraints in future implementations.

## Discussion

### Principal Findings

Our results emphasize the need to standardize mortality data collection. We developed Mali’s first electronic death certificate, aligning with WHO recommendations.

This improvement in data quantity and quality was found by Lefeuvre D et al [12] in a study on the quality of electronic and paper death certificates in France in 2010. Our results highlight challenges such as inconsistent certificates, missing forms, incomplete data, and confidentiality concerns.

The implementation of ICD-10 for coding causes of death improves data reliability and allows international comparability [13].

Reliable mortality data support effective health policies. Accurate coding aids in reducing maternal and child mortality, while the new database improves data archiving and accessibility.

Electronic data storage is undeniably better than traditional storage, optimizing record search and use [14,15]. Our structured approach incorporating an alert system and a multilevel correction and validation process, serves not only to secure data confidentiality but also to elevate the overall data quality, enabling greater precision and accuracy, all of which are part of data quality criteria [16].

Our work introduced many doctors to *ICD-10*, which is not currently part of medical education or patient records in Mali. For civil registrars, digital certificates and automatic searches improved death declarations. The statistics module in Cinz@n enables multicriteria searches and better data analysis.

From an implementation perspective, this work confirmed that successful digital health integration relies on strong institutional leadership, alignment with existing systems, and user engagement. Embedding the module within the current HIS facilitated adoption and minimized resistance, especially when paired with targeted training and ongoing support. The integration of *ICD-10* coding improved data quality and encouraged broader use of the medical record. The involvement of academic clinicians, driven by their need for structured data, further enhanced project ownership. However, limited staffing constrained timely data entry, highlighting the need to consider workforce capacity in similar initiatives. Overall, the project illustrates that a user-centered, standards-based, and system-integrated approach can enhance the adoption and sustainability of digital health tools.

### Limitations of Study

One limitation of the project is the absence of a formal long-term evaluation of its impact on the quality of mortality data

### Bests Practices and Recommendations

The lessons we have learned are

- The importance of carrying out an analysis of existing user needs as the basis for the success of such a project;
- The advantage of having pre-existing IT infrastructure;
- The academic incentives can strengthen adoption;
- The need for a clear description and definition of the role of each player in the process;
- Setting up a monitoring and steering committee, in our case the death audit committee;
- Training and support for users throughout the implementation process.

Following our work, we recommend:

- Ensuring early involvement of institutional leaders and stakeholders to build trust and alignment
- Prioritizing integration with existing systems to streamline workflows and reduce change management efforts
- Anticipating and addressing workforce constraints by exploring strategies such as task redistribution or automation.
- Investing in tailored user training and post-deployment support to facilitate adoption
- Engaging academic staff by linking system outputs with research and publication opportunities

## Supplementary material

10.2196/62949 Multimedia Appendix 1 Administrative component of the new model of death certificate.

10.2196/62949 Multimedia Appendix 2 Medical component of the new model of death certificate.

10.2196/62949 Multimedia Appendix 3 Interface to the Death Module in the Cinzan HIS.

10.2196/62949 Multimedia Appendix 4 Completing the administrative section of the death certificate.

10.2196/62949 Multimedia Appendix 5 Completing the medical section of the death certificate.

10.2196/62949 Checklist 1 iCHECK-DH checklist

## Abbreviations

HIS: Health Information System
ICD-10: International Classification of Diseases, Tenth Revision
WHO: World Health Organization
