# Supplementary figures and images for: Development of a New Electronic Death Certificate and Death Management Module Integrated Into the Health Information System of a Tertiary Hospital in Mali: Implementation Report
Source: JMIR Med Inform. 2025 Sep 18;13:e62949. doi: 10.2196/62949 (PMC12445778; doi:10.2196/62949)

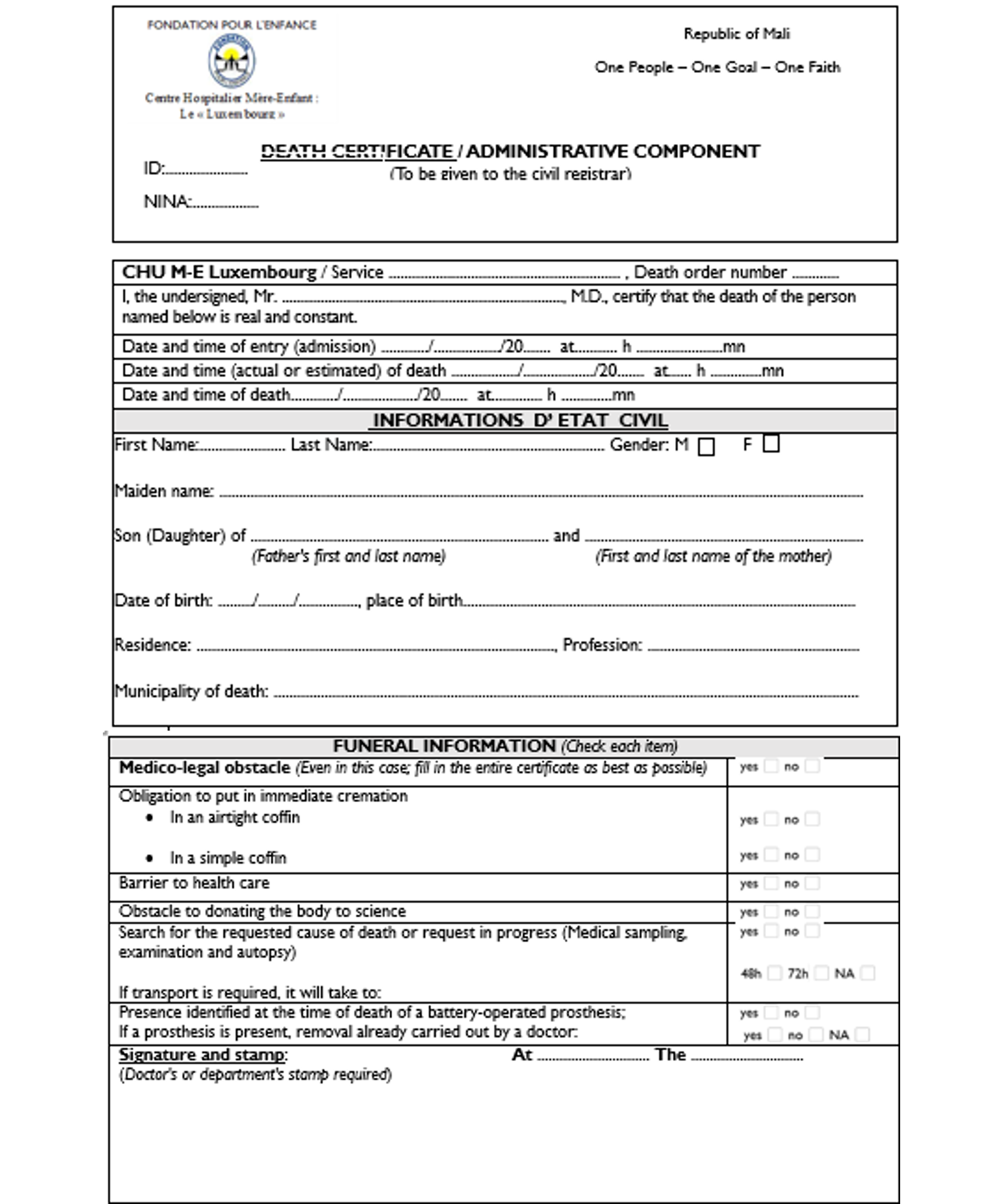

Supplement: Multimedia Appendix 1 [file medinform-v13-e62949-s001.png]

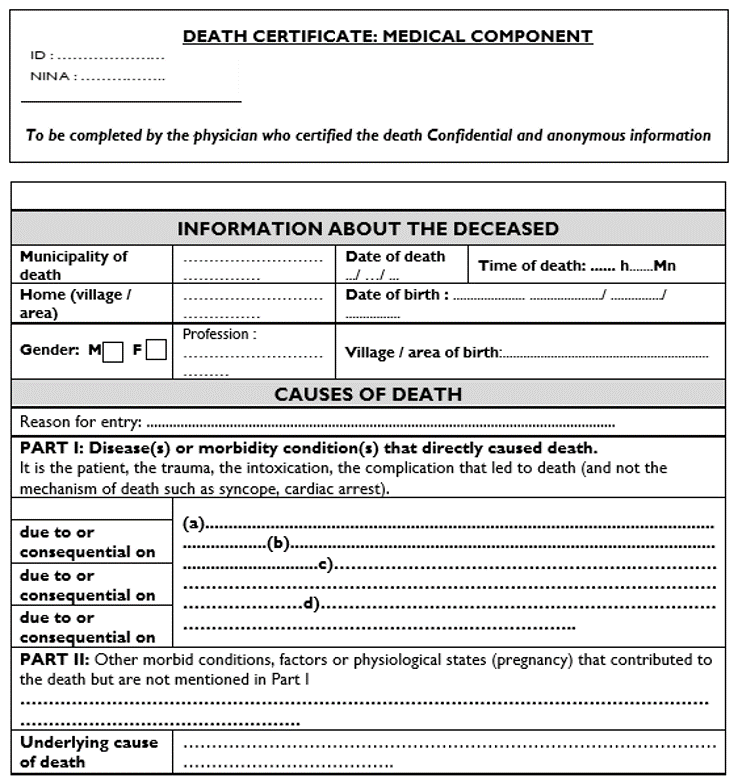

Supplement: Multimedia Appendix 2 [file medinform-v13-e62949-s002.png]

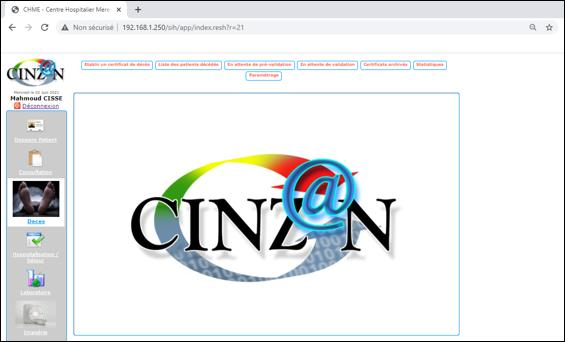

Supplement: Multimedia Appendix 3 [file medinform-v13-e62949-s003.png]

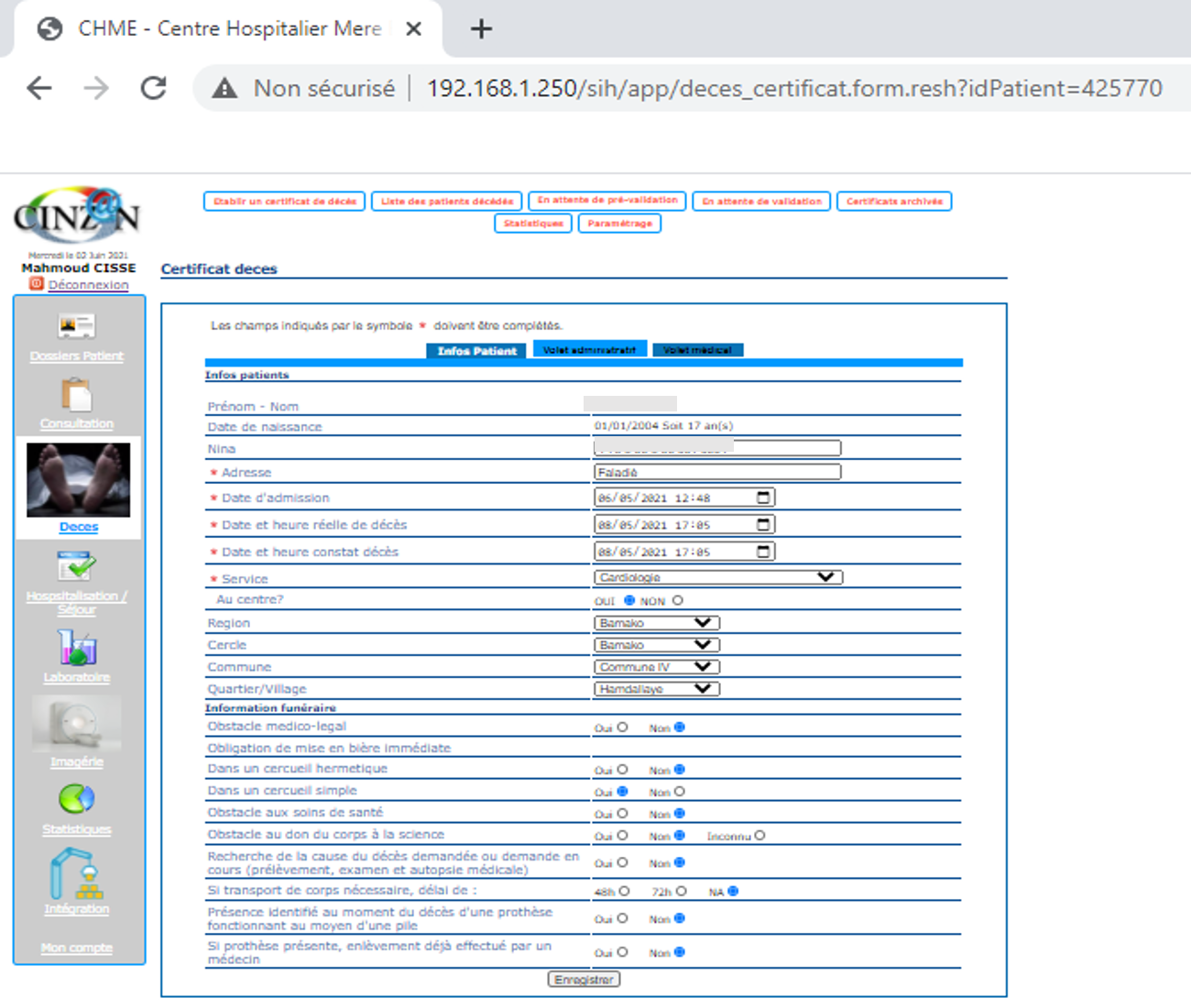

Supplement: Multimedia Appendix 4 [file medinform-v13-e62949-s004.png]

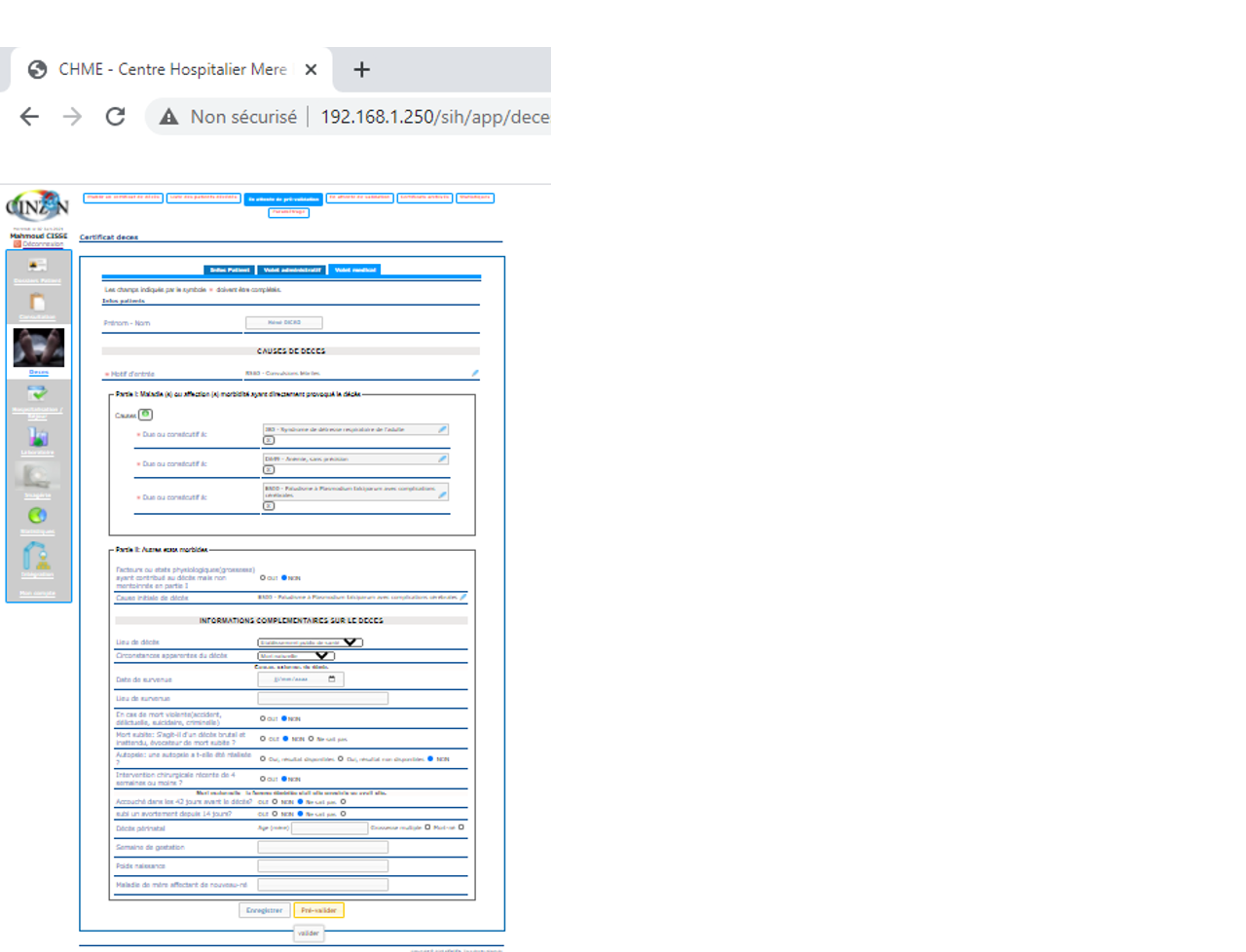

Supplement: Multimedia Appendix 5 [file medinform-v13-e62949-s005.png]
